# Attitude-Practice Gap in Ecopharmacology: A Cross-Sectional Study of Healthcare Professionals in Western Maharashtra, India

**DOI:** 10.7759/cureus.111466

**Published:** 2026-06-25

**Authors:** Shamoel Chauhan, Sujeet Divhare

**Affiliations:** 1 Department of Pharmacology, B. J. Government Medical College, Sassoon General Hospital, Pune, IND

**Keywords:** antimicrobial resistance, drug disposal, ecopharmacology, extended producer responsibility, healthcare professionals, knowledge-attitude-practice, patient counseling, pharmaceutical pollution, pharmaceutical waste management, sustainable prescribing

## Abstract

Introduction

The accumulation of active pharmaceutical ingredients (APIs) in the environment poses a serious global threat, accelerating antimicrobial resistance and disrupting ecosystems. Since standard wastewater treatment plants cannot fully degrade these pollutants, mitigating pharmaceutical wastes must begin at the source. Consequently, healthcare professionals play a critical role in prescribing sustainably and guiding patients in disposing of their medication. Given this responsibility, this study assesses and compares the knowledge, attitudes, and practices regarding ecopharmacology among different tiers of healthcare professionals and identifies specific barriers preventing sustainable clinical action.

Materials and methods

An analytical cross-sectional, observational study was conducted at a tertiary care hospital in western Maharashtra, India. The study analyzed 493 participants, including medical interns, residents, consultants, and nurses, using descriptive statistics, one-way ANOVA, a chi-square test, and multivariable logistic regression models.

Results

Despite 88.8% (n=438) of professionals recognizing pharmaceutical pollution as a severe risk, a distinct attitude-practice gap emerged. Personally, 61.0% (n=301) of participants discard leftover drugs into regular household trash. Clinically, only 22.3% (n=110) consider environmental impacts when prescribing, and only 29.0% (n=143) routinely counsel their patients on disposal. Logistic regression revealed that textbook knowledge did not predict proactive counseling. Instead, personal communication confidence is the strongest driver, explaining why experienced consultants counsel significantly more than recent graduates despite lower theoretical scores. To overcome these barriers, participants demanded systemic interventions: 91.5% (n=451) requested hospital take-back centers, 88.2% (n=435) demanded extended producer responsibility (EPR) from manufacturers, and 73.0% (n=360) requested practical continuing medical education training.

Conclusion

High environmental awareness does not organically translate into sustainable medical practice. Bridging this gap requires a systemic shift, modernizing medical education to focus on patient communication skills and establishing an accessible medication take-back infrastructure supported by industry EPR mandates.

## Introduction

The increasing levels of active pharmaceutical ingredients (APIs) in the environment have become a serious global concern, posing significant risks to public health and ecosystems [1]. Ecopharmacology examines the life cycles of chemicals of emerging concern (CECs) after they leave the clinical setting. Pharmaceuticals mainly enter the environment through two routes: the excretion of unmetabolized drugs by humans or animals and the improper disposal of unused or expired medications by consumers and institutions [2]. Once released into the environment, these molecules accumulate and have serious implications, as ecotoxicological data show endocrine disruption in aquatic species, and more concerningly, the accelerated rise of antibiotic resistance caused by continuous exposure of bacteria to low, sub-lethal concentrations of antibiotics in the environment [3].

In India, this issue is especially urgent. Being one of the world's largest manufacturers and consumers of medications, the country faces a massive challenge in managing its pharmaceutical pollution. Research studies in India revealed that standard municipal wastewater treatment facilities lack the capability or equipment to fully degrade complex APIs before releasing effluent into major river systems [4]. The region has already experienced severe ecological consequences from such pollution, including the decline and near-extinction of the indigenous *Gyps *vulture population, due to environmental exposure to diclofenac [5]. Therefore, preventing pharmaceutical pollution at its origin is the most critical strategy.

Healthcare professionals at high-prescribing institutions, such as tertiary care teaching hospitals, are essential to the success of this mitigation approach. Doctors, residents, and nurses not only determine prescription choices but also have a responsibility in guiding patients on safe disposal of medications [6]. While strict national protocols for biomedical waste within clinical settings exist, equivalent standardized regulations and public education regarding domestic medication disposal are noticeably absent.

While recent knowledge, attitude, and practices (KAP) studies have evaluated ecological awareness among healthcare workers, most research available is primarily descriptive [7]. Current studies often provide baseline statistics and treat the entire medical workforce as a single entity, ignoring the massive differences in training, generation, and responsibility from medical interns to senior consultants and nurses. It is still uncertain whether insufficient objective knowledge, lack of institutional support, or limited confidence in patient communication is the main barrier hindering healthcare providers from advising patients on safe disposal.

Ultimately, protecting the environment from pharmaceutical waste begins on the hospital floor. To identify the differences in this process, we (1) evaluated the KAP of healthcare professionals at tertiary care teaching hospitals in western Maharashtra. By (2) comparing awareness and practices across different clinical tiers, from interns to senior consultants and nurses, this study aims to (3) identify specific educational gaps and the key factors that influence a healthcare provider's likelihood to counsel patients, providing a clearer picture of where current education falls short and where future policies must adapt.

## Materials and methods

Study design, setting, and population

An analytical cross-sectional, observational study was conducted at a tertiary care hospital in western Maharashtra from January to April 2026. The study population comprised medical interns, resident doctors, consultant doctors, and nursing staff.

Sampling

**Figure 1:**
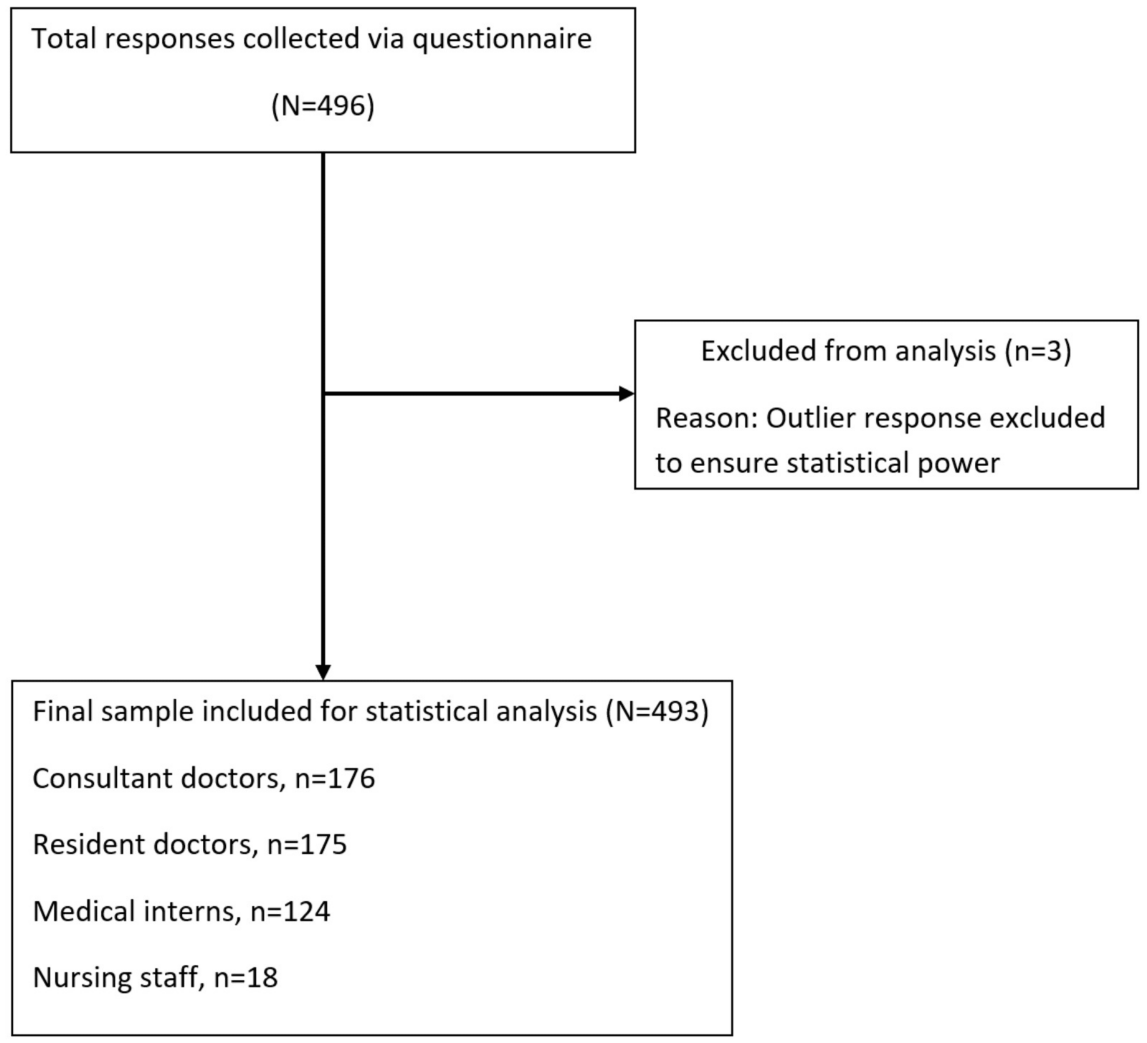
STROBE flowchart detailing participant selection and exclusion criteria STROBE: Strengthening the Reporting of Observational Studies in Epidemiology

A non-probability sampling technique (convenience method) was utilized to reach the representative population. A total of 496 participant responses were collected. Three outlier responses were excluded to ensure statistical power among the four primary subgroups, resulting in an analyzed sample of 493 participants (Figure 1).

Study instrument and data collection

Data were collected using a self-developed, structured, self-administered, English-language questionnaire. The questionnaire was created by the authors based on clinical experience and pharmacological principles. After obtaining informed consent, the questionnaire was circulated via a Google Forms link. The instrument (Appendix A) was divided into four sections.

Demographic Details

This section comprised participants' age, gender, professional role, and years of experience.

Knowledge Assessment

This includes seven objective and two subjective questions evaluating awareness of terminologies, pollution pathways, and biomedical waste segregation. Correct responses were awarded one point, for a maximum knowledge score of seven for the objective questions.

Attitude Assessment

Nine items evaluated a participant's perceptions regarding institutional responsibility, curricular reforms, and personal accountability towards pharmaceutical waste. Responses were recorded on a five-point Likert scale (ranging from 1 = strongly disagree to 5 = strongly agree), for a maximum attitude score of 45.

Practices Assessment

This includes eight categorical questions regarding the frequency of patient counseling, environmental considerations during prescribing, and personal household medication disposal habits.

To establish face and content validity, we conducted a preliminary pilot study with 20 participants. The questionnaire was refined using their feedback, and these responses were excluded from the final data analysis.

Data analysis

All collected data were entered into a Microsoft Excel spreadsheet (Microsoft Corporation, Redmond, WA). A total of 493 participants satisfactorily completed the questionnaire and were included in the study evaluation. The data were cleaned, coded, and analyzed using R software (version 4.4.3; R Foundation for Statistical Computing, Vienna, Austria). Descriptive statistics were used to summarize demographic variables and baseline practice responses. We utilized a one-way ANOVA to evaluate differences in total knowledge and attitude scores among the four groups, followed by a Tukey post-hoc test to identify specific group variations. For the categorical questions about daily clinical habits, we used the chi-square test to find associations between a participant's profession and their actions. Finally, we used a multivariable logistic regression model to identify the strongest predictor of good clinical practice, looking at whether age, knowledge, or personal confidence is what actually prompts a healthcare worker to counsel a patient.

Across all statistical tests, a p-value of < 0.05 was considered statistically significant.

Ethical considerations

The study protocol was approved by the Institutional Ethics Committee (Certificate number: BJGMC/IEC/Pharmac/ND-0126007-007). All participants provided electronic informed consent. Participation was voluntary, and confidentiality was maintained.

## Results

Demographic characteristics

A total of 493 healthcare professionals were analyzed (Table 1). The study population was highly diverse in its clinical structure, with the majority comprising actively practicing consultant doctors, followed by resident doctors, medical interns, and nursing staff. The study group had an average age of 35.3 years, a nearly equal gender distribution, and spanned varying levels of clinical experience, thereby providing a complete representation of the hospital workforce.

**Table 1:** Baseline demographic and professional characteristics of the study population

Characteristic	Category	Total sample (N=493)
Age (years)						
	Mean ± SD	35.3 ± 12.7
	Range	22-74
Gender, n (%)					
	Male	252 (51.1%)
	Female	240 (48.7%)
	Prefer not to say	1 (0.2%)
Professional role, n (%)				
	Consultant doctor	176 (35.7%)
	Resident doctor	175 (35.5%)
	Medical intern	124 (25.2%)
	Nursing staff	18 (3.7%)
Years of clinical experience, n (%)			
	Less than 1 year	134 (27.1%)
	1-5 years	167 (33.9%)
	6-10 years	29 (5.9%)
	More than 10 years	163 (33.1%)

Objective knowledge on ecopharmacology

**Figure 2:**
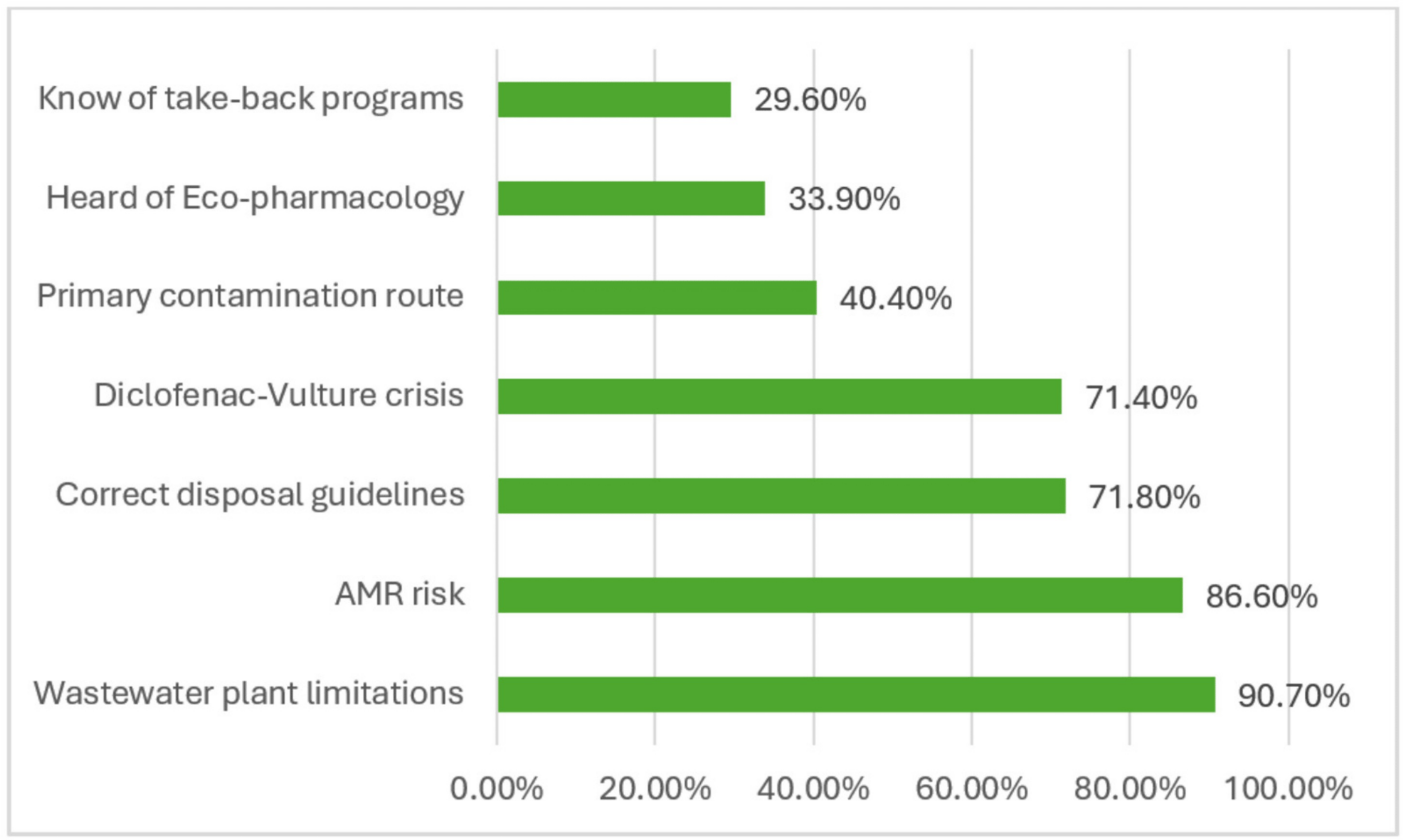
Awareness of macro-ecological impacts and mitigation approaches among healthcare professionals AMR: Acceleration of Antimicrobial Resistance

The assessment of objective knowledge showed a significant gap between the understanding of theoretical environmental impacts and practical mitigation approaches (Figure 2).

Participants showed different levels of knowledge regarding common disposal methods and contamination pathways. While the recommended household disposal method (mixing unappealing substances with medications before trashing) was correctly identified by 71.8% (n=354) of respondents, there was confusion about how pharmaceuticals enter the ecosystem. Only 40.4% (n=199) correctly identified human and animal excretion via the sewage system as the primary route of environmental contamination. Awareness of institutional solutions was notably low; only 33.9% (n=167) of healthcare professionals were familiar with the term "ecopharmacology", and even fewer (29.6%, n=146) were aware of official medication "take-back" programs.

Ecopharmacological practices and disposal habits

**Figure 3:**
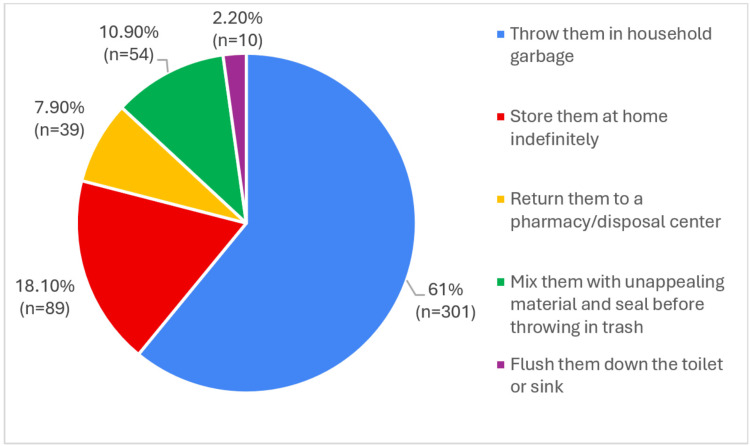
Personal household medication disposal practices

Evaluating routine habits both at home and in the clinic showed significant differences in how sustainable practices were actually being applied. In their private lives, the majority of participants (61%, n=301) admitted to throwing leftover or expired medications into regular household trash, while 18.1% (n=89) kept such medications at home indefinitely. Only 7.9% (n=39) reported ever returning unused or expired drugs to a pharmacy or designated hospital disposal facility (Figure 3).

**Figure 4:**
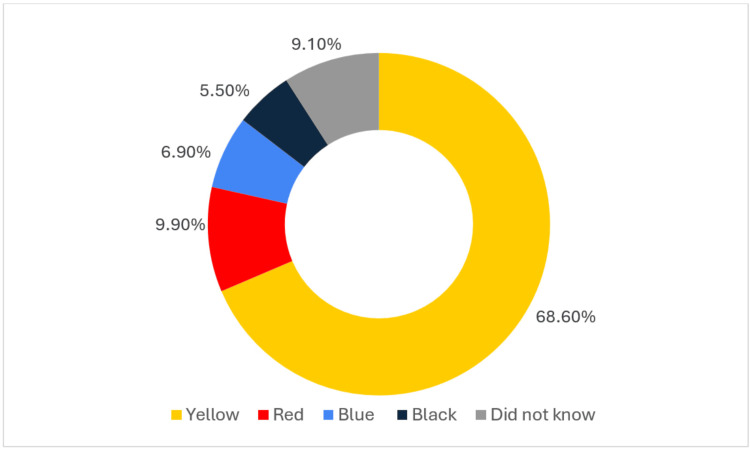
Knowledge of institutional biomedical waste segregation protocols for expired pharmaceuticals

To evaluate how these personal practices translated to the clinical workspace, participants were posed an objective knowledge question regarding institutional waste segregation (Figure 4). According to India's Bio-Medical Waste Management rules (2016), expired or discarded pharmaceuticals must be disposed of in yellow-colored containers [8].

Although 68.6% (n=338) correctly identified the yellow bin as the mandated container for expired pharmaceuticals, close to 30% (n≈148) either answered incorrectly, selecting red, blue, or black bins or remained unsure, demonstrating a need for further training on the hospital floor.

These practical gaps appear to stem from a lack of foundational training; only 32.7% (n=161) of respondents reported receiving any formal education on safe pharmaceutical disposal during their medical or nursing training. When asked to assess their own practices, more than half (54.1%, n=267) acknowledged their routines did not follow sustainable principles, and only a small fraction (5.9%,* *n=29) believed they were fully adhering to them.

The attitude paradox

**Figure 5:**
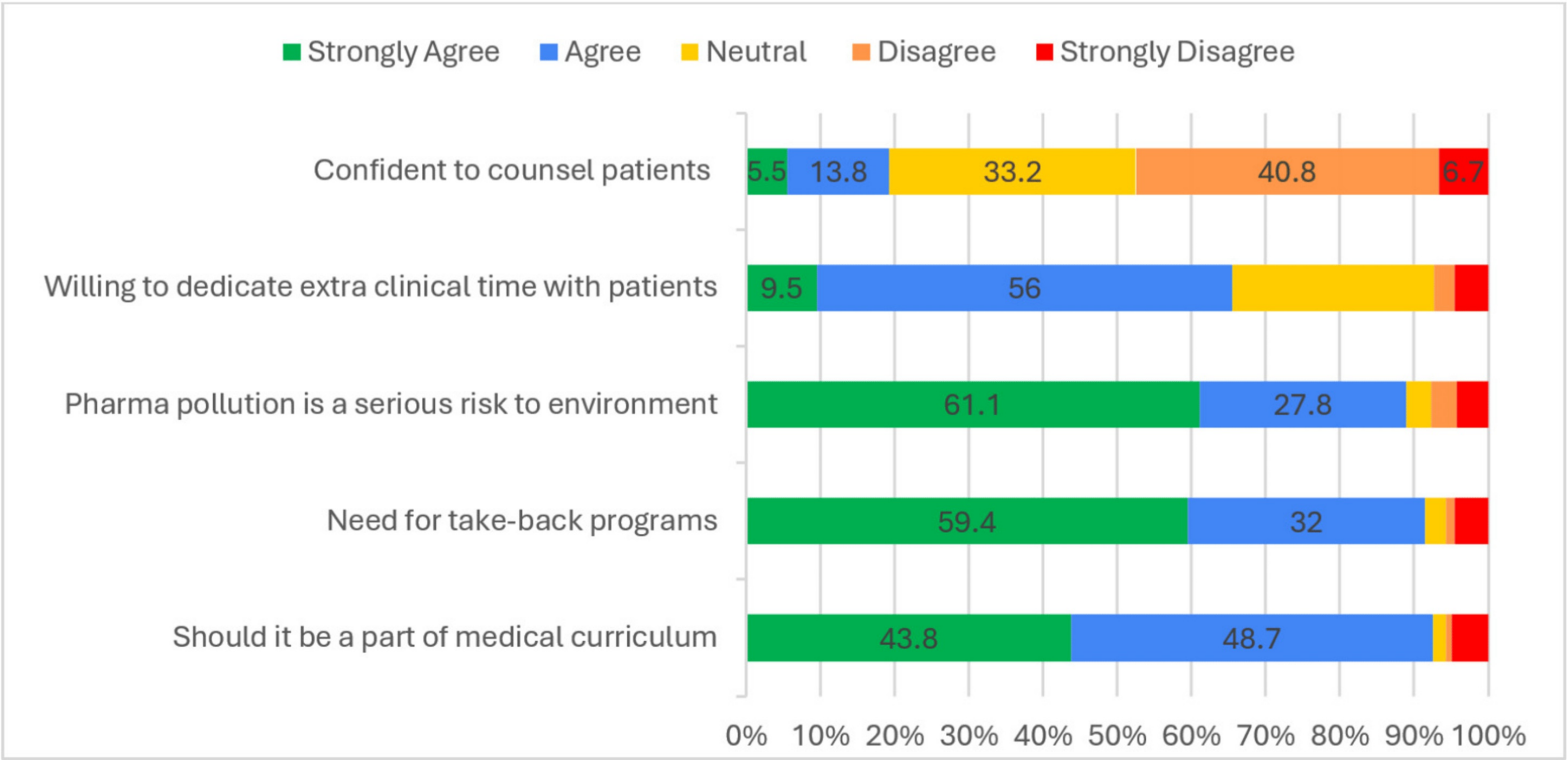
Attitudes and perceptions of healthcare professionals toward environmental responsibility and systemic interventions

Despite gaps in formal ecopharmacological knowledge and sustainable disposal practices, the surveyed group showed positive attitudes towards environmental responsibility (Figure 5).

Overall, 88.8% (n=438) of healthcare professionals agreed or strongly agreed that pharmaceutical pollution is a severe risk to the environment, and 91.4% (n=451) believed that hospitals should implement medication take-back centers. Additionally, 92.5% (n=456) supported making ecopharmacology a required part of medical education. On a personal level, 65.5% (n=323) were willing to spend extra time during patient visits to discuss these concerns.

**Figure 6:**
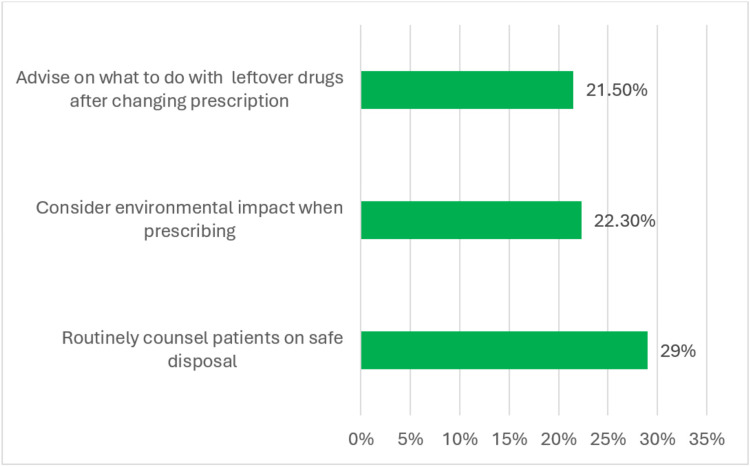
Routine clinical practices and frequency of patient counseling on sustainable drug disposal

However, theoretical willingness did not translate into clinical practice (Figure 6).

Only 29.0% (n=143) reported regularly advising patients on how to safely dispose of leftover or unused medications; 22.3% (n=110) routinely consider a drug's environmental impact when prescribing, and a mere 21.5% (n=106) advise patients on what to do with leftover medications when changing prescriptions.

The gap between theoretical willingness to act (65.5%, n=323) and actual patient counseling (29.0%, n=143) appears to stem from a lack of personal readiness. When asked to assess their own communicative readiness, only 19.3% (n=95) of healthcare providers felt confident in their knowledge to counsel patients effectively. This suggests that, while a desire to practice sustainably exists, the main barrier lies in a lack of confidence in communicating these details.

Comparative analysis across professional groups

We compared the objective knowledge, subjective attitudes, and clinical habits of the four professional groups to identify specific gaps in knowledge and practices across different roles (Table 2).

**Table 2:** Comparative analysis of knowledge scores, attitude perceptions, and daily clinical practices among different healthcare professional tiers * One-way ANOVA; † Includes participants who responded "always," "often," or "sometimes"; ‡ Chi-square test

Characteristic	Consultant doctor (n=176, 35.7%)	Resident doctor (n= 175, 35.5%)	Medical intern (n=124, 25.2%)	Nursing staff (n=18, 3.7%)	p-value
Total knowledge score (Mean ± SD)	4.27 ± 1.34	5.89 ± 1.23	5.71 ± 1.09	5.33 ± 1.41	<0.001*
Total attitude score (Mean ± SD)	34.61 ± 9.55	36.05 ± 3.15	35.02 ± 2.07	32.94 ± 9.99	0.076*
Considers environmental impact while prescribing, n (%)^†^	81 (46%)	19 (10.9%)	3 (2.4%)	7 (38.9%)	<0.001^‡^
Proactively counsel patients on safe disposal, n (%)^†^	46 (26.1%)	25 (14.3%)	3 (2.4%)	12 (66.7%)	<0.001^‡^

A one-way ANOVA showed a statistically significant difference in overall knowledge scores across the professional roles (F=57.79; p<0.001). Tukey's post-hoc test revealed that consultant doctors scored significantly lower than both resident doctors (p<0.001) and medical interns (p<0.001). This points towards a deficit in ecopharmacology training among more experienced clinicians, while recent graduates appear better informed.

In contrast, no significant differences emerged in overall attitude scores across the roles (F=2.31; p=0.076). All groups showed a comparably high degree of environmental concern and institutional expectation, indicating that a lack of motivation or concern is not the primary barrier for senior staff.

When evaluating daily clinical habits, consultant doctors factored environmental impacts into their prescriptions significantly more than junior doctors, despite having lower theoretical knowledge scores (chi-square test, χ2=101.49; p<0.001). Similarly, consultant doctors and nursing staff reported advising patients on proper drug disposal significantly more often than residents or interns (χ2=60.17; p<0.001), indicating that theoretical knowledge does not translate into clinical practice among younger doctors.

Predictors of proactive patient counseling

To understand what actually motivates healthcare professionals to advise patients on safe medication disposal, we conducted a multivariable logistic regression analysis (Table 3).

**Table 3:** Multivariable logistic regression identifying independent predictors of patient counseling on ecopharmacology * Indicates statistical significance at p<0.05. Proactive patient counseling compared participants who answered "Always," "Often," and "Sometimes" versus those who did not.

Predictor variable	Odds ratio (OR)	95% confidence interval	p-value
Age (continuous)	1.03	1.00-1.050	0.020*
Objective knowledge score (out of 7)	1.03	0.84-1.25	0.798
Personal communication confidence (1-5 scale)	2.37	1.81-3.09	<0.001*

This allowed us to assess three key factors: age, objective knowledge on ecopharmacology, and personal confidence in communication to determine their impact on clinical behavior.

The most notable finding was that factual knowledge had no significant effect on practice (p=0.798). Even after adjusting for other variables, having a stronger textbook understanding of environmental pharmacology did not increase the likelihood that a professional would provide disposal guidance.

Instead, personal confidence in communication proved to be the strongest predictor. For every level of increase in a professional's personal confidence in communication, they were more than twice as likely to proactively counsel a patient on disposal (odds ratio (OR)=2.37; p<0.001). Increasing age also appeared as an independent positive predictor of initiating such discussions (p=0.020).

These findings clarify the behavioral paradox observed among senior professionals: senior staff are not advising patients more due to greater ecopharmacological expertise, but rather because their years of experience have built confidence in managing patient conversations.

## Discussion

A key finding of this study is the clear disconnect between strong theoretical awareness of environmental issues and actual clinical practice. Although there is widespread agreement that pharmaceutical pollution, especially its role in driving antimicrobial resistance, represents a serious environmental concern, this understanding has not led to sustainable personal disposal habits or proactive patient counseling. Most clinicians do not routinely factor environmental consequences into their prescribing decisions, indicating that recognizing the dangers of prolonged exposure to sub-lethal pharmaceutical contaminants does not necessarily make them act on that knowledge [3]. As a result, even trained healthcare providers often resort to improper disposal methods similar to those seen across the general population [6], revealing a shortcoming in integrating environmental awareness into routine clinical workflow.

This behavioral paradox stems from an incomplete understanding of applied ecopharmacology. While participants were generally aware of major historical ecological events such as the vulture population decline caused by diclofenac, they showed a lack of knowledge of smaller-scale, everyday processes. Less than half recognized that human excretion is the primary pathway for active pharmaceutical ingredients to enter water systems [9], and nearly a third failed to correctly identify the mandated yellow container for expired drug segregation. This suggests a training gap that favors historical ecotoxicology rather than practical sustainable practices. This shortfall in applied education is mirrored in the recent evaluations of tertiary care centers, which confirmed that, while frontline healthcare workers possess high practical awareness of standard infectious waste, their knowledge regarding chemical and pharmaceutical waste protocols lags significantly behind [10].

Beyond knowledge deficits, the data reveal a generational paradox in clinical settings. Although younger medical staff, interns, and junior residents performed better on theoretical ecopharmacology due to recent updates to medical curricula, this textbook knowledge rarely converted to effective patient counseling. Conversely, senior consultants, despite lower scores on factual knowledge questions, were more likely to proactively advise patients about environmental impacts. This suggests that theoretical knowledge is not the primary driver of sustainable practice; rather, clinical communication is key. While traditional Indian medical education successfully imparts clinical facts, it often leaves young doctors unprepared for patient conversations, leaving a critical communication gap [11]. Ultimately, it is the accumulated experience, conversational skill, and self-efficacy built through years of hands-on experience that empowers a physician to confidently guide patients on sustainable disposal [12].

Beyond individual communication barriers, participant attitudes point toward profound systemic limitations. Most participants acknowledged that addressing pharmacological pollution requires coordinated action across multiple sectors. Although nearly all (91.1%) stressed the importance of increasing public awareness, they also noted that such efforts are ineffective without strong institutional frameworks to support them. A majority (65.3%) identified the healthcare sector as essential in driving sustainable practices, while an even larger proportion (88.2%) demanded concurrent support from pharmaceutical manufacturers through extended producer responsibility (EPR) mandates. This perspective highlights a major challenge: even the most environmentally conscious clinicians in India face limitations due to a lack of infrastructure. Consequently, 91.5% of participants called for the establishment of medication take-back centers. Regional assessments of tertiary care facilities in India support this need, highlighting that returning unused medications directly to the manufacturer, the core mechanism of EPR, is the most practical solution to the growing problem of pharmaceutical waste [13]. Ultimately, localized public health research clearly indicates that until physical, accessible take-back programs are established, safely disposing of medications will continue to be a challenge for healthcare workers and the public [14].

To effectively solve these systemic barriers, we must reconsider how we are educating our healthcare professionals from the ground up. There is a clear consensus among clinicians that their current education is deficient; our data show that 92.5% of respondents want ecopharmacology to be a mandatory part of the medical curriculum. To bridge the gap among older, experienced professionals, 73.0% of the participants also agreed on attending continuing medical education (CMEs) focused on sustainable prescribing. However, the answer is not just adding textbook material or passive lectures on practices. What is required is a move towards active, skills-based learning. Evidence from real-world teaching studies supports this approach; switching from traditional lectures to interactive, student-centered simulations led to improvements in biomedical waste management practices on the clinical floor [15]. By incorporating these practical, point-of-care simulations into both medical schools and CME programs, we can give clinicians the tools needed to transform environmental awareness into consistent daily clinical practice [16]. Until we can make a systemic shift in terms of hands-on learning and EPR- backed hospital infrastructure, healthcare workers will continue to remain in the paradox, deeply wanting to protect the environment but lacking the practical tools and institutional support to do so.

Strengths and limitations

A primary strength of this study is its large, diverse sample size (N = 493), encompassing multiple levels of the clinical workforce. This diversity allowed for a detailed comparative analysis that uncovered distinct generational differences. Rather than just blaming healthcare workers for poor disposal habits, this study highlights the real barriers they face. These findings offer policymakers highly practical, real-world data in a critical yet underexplored field of Indian healthcare.

However, certain limitations must be acknowledged. First, the cross-sectional design captures data at a single time point; therefore, while predictors of sustainable practice were identified, strict long-term causality cannot be established. Second, reliance on self-reported questionnaires introduces an inherent risk of social desirability bias, where participants may overstate their eco-friendly attitudes to align with professional behavior. Third, although the overall cohort is large, the relatively small representation of nursing staff (n = 18) limits the statistical power when drawing definitive, subgroup-specific conclusions for this professional group. Additionally, because we used convenience sampling, we did not calculate the regression sample size in advance. Despite this, the model proved highly stable, and our analysis achieved an events per variable ratio of 47.7, comfortably exceeding standard statistical requirements. Finally, while the dataset is statistically strong, it was collected from a specific regional tertiary care center. While this provides a strong baseline, it may not fully reflect the day-to-day challenges faced in other parts of the country.

## Conclusions

This study emphasizes an important insight: awareness of pharmaceutical pollution alone does not translate into medical practice. The gap between healthcare professionals' environmental concerns and their actual disposal habits is not due to a lack of caring. Rather, it stems from a lack of confidence in patient communication and the absence of practical systems to support responsible disposal. We must stop placing the burden of environmental stewardship on healthcare professionals alone; instead, mitigating this crisis requires a systemic shift. Medical education must evolve, swapping passive textbook lectures for practical, hands-on training that prepares providers for real-world discussions. Simultaneously, policymakers should implement EPR regulations to establish convenient medication take-back centers in hospitals across India. Meaningful change will only occur when our clinicians are supported by both conversational strategies and institutional backing, enabling environmental awareness to become a routine clinical practice.

## Appendices

Appendix A: Questionnaire

1. I have read the above information, and I consent to participate in this study.

A) Yes, I consent

B) No, I do not consent

Demographic Characteristics

2. Age: _____ (in years)

3. Gender:

A) Male

B) Female

C) Prefer not to say

D) Other: ______(please specify)

4. What currently best describes your professional role?

A) Medical intern

B) Resident Doctor

C) Nurse/ Nursing staff

D) Faculty/ Consultant Doctor

5. Your primary department/ Specialty?

A) Medicine & Allied (e.g. Dermatology, Psychiatry, etc.)

B) Surgery & Allied (e.g. Orthopedics, Anesthesia, etc.)

C) Obstetrics & Gynecology or Pediatrics

D) Pre/Para-clinical subject

E) Nursing staff

6. How many years of professional experience do you have in the healthcare field?

A) Less than 1 year

B) 1-5 years

C) 6-10 years

D) More than 10 years

Knowledge Assessment

7. Have you ever heard the term 'Ecopharmacology' or 'Environmental Pharmacology', or 'Green Pharmacy'?

A) Yes

B) No

8. Which of the following best defines "Ecopharmacology/Green Pharmacy"?

A) A pharmacy that primarily sells herbal or ayurvedic medicines

B) An approach to minimize the environmental impact of drugs throughout their lifecycle

C) A government initiative to provide subsidized (low-cost) medicines

D) I don't know

9. What is the most significant route for pharmaceuticals to enter the environment?

A) Accidental spills during drug manufacturing

B) Improper disposal of unused/expired medicines in the trash

C) Human/Animal excretion (via urine and feces) into the sewage system

D) None of the above

10. Do you think wastewater treatment plants in most Indian cities are designed to effectively remove all pharmaceutical residues?

A) Yes

B) No

C) I don't know

11. Which of the following is considered a major long-term public health consequence of pharmaceutical residues (specifically antibiotics) in the environment?

A) Increased risk of non-communicable diseases

B) Acceleration of Antimicrobial Resistance (AMR)

C) Bioaccumulation in the human body

D) Soil erosion

12. By which widely used drug in India was the decline of the vulture population caused by environmental contamination?

A) Paracetamol

B) Metformin

C) Diclofenac

D) Ivermectin

13. Do you know whether your hospital or community has a drug disposal or take-back program?

A) Yes

B) No

C) I don't know

14. What is the most environmentally sound way for a patient to dispose of unused/expired tablets at home, if a take-back program is unavailable?

A) Flush them down the toilet

B) Crush or break the medicines and bury them in the soil

C) Mix them with unappealing substances, seal them, and throw them together with the household trash

D) Separate the medicine, dissolve it in water, and pour it into the drain

15. According to India's Biomedical Waste Management Rules, which color-coded bin is designated for expired and cytotoxic drugs?

A) Yellow bin

B) Blue bin

C) Red bin

D) Black bin

E) I don't know

Attitude Assessment

16. Please indicate your level of agreement with the following statements.

Responses are recorded on a five-point Likert scale:

1 = Strongly disagree, 2 = Disagree, 3 = Neutral, 4 = Agree, 5 = Strongly agree.

A) Pharmaceutical waste from our hospital poses a significant risk to the environment.

B) Teaching of environmental pharmacology should be included in the medical/nursing curriculum.

C) It is the responsibility of all healthcare professionals to reduce pharmaceutical pollution.

D) I feel confident in my knowledge to answer patient questions about drug disposal.

E) I am willing to spend extra time during patient consultations to discuss medication disposal.

F) My institution should establish a "Drug Take-Back/Collection Box" for patients to safely dispose of their unused medicines.

G) There should be public awareness campaigns on proper drug disposal.

H) I would willingly dedicate time to attend training/CME sessions focused solely on safe drug disposal practices.

I) The pharmaceutical industry should be held more responsible for the environmental fate of its products.

Practices Assessment

17. Have you ever been educated about proper medicine disposal during your training?

A) Yes

B) No

18. In your personal life, how do you usually dispose of expired or leftover medicines at home?

A) Throw them in the household garbage

B) Flush them down the toilet or sink

C) Return them to a pharmacy/disposal center

D) I store them at home indefinitely

E) Mix with unappealing material and seal before throwing it in the trash

19. Have you ever returned expired/unused medicines to a pharmacy or hospital?

A) Yes

B) No

20. In your clinical practice, do you counsel patients on the safe disposal of their unused/leftover medications?

A) Always

B) Sometimes

C) Rarely

D) Never

E) Only if the patient asks

21. When a patient's prescription is changed, how often do you advise them on what to do with the previously prescribed, unused medication?

A) Always

B) Often

C) Sometimes

D) Rarely

E) Never

F) Only if the patient asks

22. In your hospital ward/clinic area, are you aware of a designated separate bin/protocol specifically for the disposal of expired or partially used medications?

A) Yes

B) No

C) Unsure

23. Do you personally consider environmental impact when choosing or recommending a drug (e.g., antibiotics, hormones, NSAIDs)?

A) Always

B) Sometimes

C) Rarely

D) Never

24. Do you believe your current practice aligns with principles of Ecopharmacology/Green Pharmacy?

A) Yes

B) Partially

C) No

25. If a patient asks you for a safe way to dispose of their unused medication at home, what specific action would you recommend? _________(Open-ended response)
